# Current Views on Pathophysiology and Potential Therapeutic Targets in Sjögren’s Syndrome: A Review from the Perspective of Viral Infections, Toll-like Receptors, and Long-Noncoding RNAs

**DOI:** 10.3390/jcm12185873

**Published:** 2023-09-10

**Authors:** Yoshiro Horai, Toshimasa Shimizu, Masataka Umeda, Shin-Ya Nishihata, Hideki Nakamura, Atsushi Kawakami

**Affiliations:** 1 Department of Rheumatology, Sasebo City General Hospital, Sasebo 857-8511, Japan; 2 Department of Immunology and Rheumatology, Division of Advanced Preventive Medical Sciences, Nagasaki University Graduate School of Biomedical Sciences, Nagasaki 852-8523, Japan; toshimasashimizu2000@yahoo.co.jp (T.S.); masatakau0807@nagasaki-u.ac.jp (M.U.); atsushik@nagasaki-u.ac.jp (A.K.); 3 Clinical Research Center, Nagasaki University Hospital, Nagasaki 852-8501, Japan; 4 Department of Rheumatology, National Hospital Organization Ureshino Medical Center, Ureshino 843-0393, Japan; nissyhatter24@gmail.com; 5 Division of Hematology and Rheumatology, Department of Medicine, Nihon University School of Medicine, Tokyo 173-8610, Japan; nakamura.hideki@nihon-u.ac.jp

**Keywords:** interferon, JAK/STAT pathway, long-noncoding RNA, Sjögren’s syndrome, toll-like receptor, virus

## Abstract

Sjögren’s syndrome (SS) is a rheumatic disease characterized by sicca and extraglandular symptoms, such as interstitial lung disease and renal tubular acidosis. SS potentially affects the prognosis of patients, especially in cases of complicated extraglandular symptoms; however, only symptomatic therapies against xerophthalmia and xerostomia are currently included in the practice guidelines as recommended therapies for SS. Considering that SS is presumed to be a multifactorial entity caused by genetic and environmental factors, a multidisciplinary approach is necessary to clarify the whole picture of its pathogenesis and to develop disease-specific therapies for SS. This review discusses past achievements and future prospects for pursuing the pathophysiology and therapeutic targets for SS, especially from the perspectives of viral infections, toll-like receptors (TLRs), long-noncoding RNAs (lncRNAs), and related signals. Based on the emerging roles of viral infections, TLRs, long-noncoding RNAs and related signals, antiviral therapy, hydroxychloroquine, and vitamin D may lower the risk of or mitigate SS. Janus-kinase (JAK) inhibitors are also potential novel therapeutic options for several rheumatic diseases involving the JAK-signal transducer and activator of transcription pathways, which are yet to be ascertained in a randomized controlled study targeting SS.

## 1. Introduction

Sjögren’s syndrome (SS) is a rheumatic disease characterized by sicca symptoms due to lymphocytic infiltration of the exocrine glands and the emergence of autoantibodies such as anti-Ro/SS-A and La/SS-B antibodies.Anti-Ro/SS-A and La/SS-B antibodies are crucial items for the diagnosis of SS adopted in the 2002 American-European Consensus Group SS classification criteria [1] as well as pathogenic in neonatal lupus [2]. Although the pathogenesis of SS is yet to be elucidated, genetic and environmental factors, including viral infections, are presumed to be responsible in its development [3]. SS is generally considered a disease of dry eyes and mouth and is not necessarily fatal; however, in addition to sicca symptoms, extraglandular symptoms such as interstitial lung disease and renal tubular acidosis might complicate it, the degree of which is reflected in the European Alliance of Associations for Rheumatology (formerly European League Against Rheumatism) SS Disease Activity Index (ESSDAI) [4]. Prognosis of patients with SS may depend on its clinical disease activity measured by ESSDAI: patients with SS of ESSDAI of 14 or more were reported to have a higher mortality [5]. In addition to the overall ESSDAI, each component included in the ESSDAI could affect the prognosis of patients with SS. It was recently reported that patients with SS and hypergammaglobulinemia in a Chinese population showed a significantly higher mortality rate compared to patients without hypergammaglobulinemia, and serum immunoglobulin G (IgG) levels were positively correlated with the risk of death [6]. Therefore, early diagnosis, assessment of systemic symptoms, and treatment should be implemented to improve not only the quality of life but also the prognosis of patients with SS.

SS is presumed to be a multifactorial disease; therefore, a multidisciplinary approach is necessary to clarify the whole picture of the pathogenesis of SS and to develop disease-specific therapy against SS. Molecularly targeted therapies, including biological disease-modifying anti-rheumatic drugs (bDMARDs) and Janus kinase inhibitors (JAKis) have revolutionized the treatment of rheumatic diseases such as rheumatoid arthritis (RA) and systemic lupus erythematosus (SLE) [7]. However, only symptomatic therapies such as tear drops and muscarinic agonists are currently included in practice guidelines as recommended therapies for SS [8,9], and effects of bDMARDs such as belimumab and abatacept for SS are yet to be confirmed by larger randomized controlled trials [10]: bDMARDs were administered to patients with other rheumatic diseases, where SS was a secondary complication [11]. The clinical application of molecular targeted drugs for SS is yet to be established.

In this review, we discuss past achievements and future prospects for pursuing the pathophysiology and therapeutic targets for SS, especially from the perspectives of viral infections, toll-like receptors (TLRs), long-noncoding RNAs (lncRNAs), and related signals. SS is classified into primary SS and secondary SS (sSS) based on the presence or absence of other rheumatic diseases, such as RA and SLE. The diagnosis of primary SS is made based on different criteria from sSS in the 2002 American-European Consensus Group SS classification criteria [1], and sSS is renamed as associated SS [12]. Hereafter, we refer to primary SS as SS.

## 2. Role of Viral Infections in SS

As mentioned in the introduction, viral infections are believed to be involved in the pathogenesis of SS. Similar to other autoimmune diseases, molecular mimicry and structural similarities between viral proteins and proteins in the human body, which lead to autoimmune responses, are assumed in the pathogenesis of SS [3]. In addition to molecular mimicry, bystander activation and epitope spreading are also assumed in the pathogenesis of SS [3].

We previously reported a high positivity rate of anti-human T-cell leukemia virus type I (HTLV-I) antibody in patients with SS and higher complication rates of extraglandular manifestations, such as uveitis, myopathy, and recurrent pyrexia, in patients that were SS positive for anti-HTLV-I antibody than in those negative for anti-HTLV-I antibody [13]. Subsequently, we found a high prevalence of HTLV-I infection in patients with SS compared to the general population in Nagasaki Prefecture, western Japan [14]. We also found that the prevalence of rheumatoid factor, antinuclear antibody, and anti-Ro/SS-A antibody did not differ among patients with HTLV-I-associated myelopathy with SS, SS positive for the anti-HTLV-I antibody, and SS negative for the anti-HTLV-I antibody [15]. We also reported HTLV-I bZIP factor-mediated forkhead box P3 expression in labial salivary glands (LSGs) of patients with HTLV-I-seropositive SS [16].

In addition to HTLV-I, Epstein–Barr, human immunodeficiency, cytomegalo, and hepatitis viruses have been reported to participate in the pathophysiology of SS [3]. Yeh et al. analyzed the association between chronic hepatitis and SS. Chronic hepatitis B virus (HBV) infection was associated with an increased risk of SS in men. However, hepatitis C virus (HCV) was associated with SS in both genders [17]. It should be noted that active HCV infection was an exclusion criterion in the 2016 American College of Rheumatology/European League Against Rheumatism (currently European Alliance of Associations for Rheumatology) classification criteria for SS [18]. Severe acute respiratory syndrome coronavirus 2 (SARS-CoV-2) has been a global concern since the 2020s. It is not clear whether coronavirus disease- 2019 (COVID-19) is an onset factor of SS: there is currently only one case report that described a patient with increasing titers of anti-Ro/SS-A and La/SS-B antibody and salivary gland (SG) biopsy compatible with SS and confirmed subsequently COVID-19 [19].

## 3. Roles of TLRs and Their Downstream Mediators in SS

Innate immunity plays an important role in the defense mechanisms against viral infections. TLRs play a central role in innate immunity by detecting pathogen-associated molecular patterns derived from viruses. In addition, TLRs are engaged in initiating bystander T cell activation [20]. Many kinds of viruses, including the aforementioned Epstein–Barr and human immunodeficiency viruses, have been reported to trigger TLR-mediated innate immune responses, leading to the production of proinflammatory cytokines and type I interferon (IFN)s in response to the recognition of ligands derived from viruses [21]. Type I IFNs induced by TLR signaling would be also involved in the production of autoantibodies; patients positive for anti-Ro/SS-A antibodies more often have higher IFN-α scores [22]. Considering the above discussion on viral infections in the pathophysiology of SS, TLRs are likely to be associated with the pathophysiology of SS.

We explored the pathophysiology of SS from the perspective of innate immunity in the SGs, particularly TLR-related apoptosis. We noticed TLRs 2–4, which participate in innate immune responses by recognizing various pathogens, including viruses and bacteria, are strongly expressed not only in infiltrating mononuclear cells but also in ductal and acinar epithelial cells in the LSGs of patients with SS [23]. TLR3, which recognizes double-stranded RNA, has been reported to induce B cell-activating factors in SG epithelial cells [24]. We showed that TLR3-mediated apoptosis via the phosphatidylinositol 3-kinase-Akt (protein kinase B) signaling pathway was induced by stimulation of polyinosinic-polycytidylic acid (poly I:C), a synthetic analog of double-stranded RNA [25]. Next, we investigated the downstream molecules of TLR3. In the SGs affected with SS, rabbit anti-receptor-interacting serine/threonine protein kinase was mildly stained in the ducts and alveoli, whereas neither phosphorylated Fas-associated protein with the death domain nor cleaved-caspase 8 was detected. Poly I:C stimulation induced increased expression of receptor-interacting serine/threonine protein kinase, phosphorylated Fas-associated protein with death domain, and cleaved-caspase 8. TLR3 expression was also increased with poly I:C stimulation; triggering of TLR3 induced the opposite phenotypes to the unstimulated SG epithelial cells, suggesting the presence of humoral factors interfering with TLR3-mediated effects in vivo. An apoptosis antibody assay in the presence or absence of epidermal growth factor, which has been shown to exert anti-apoptotic effects through the activation of phosphatidylinositol 3-kinase-Akt and nuclear factor kappa B [26], revealed increased expression of hemeoxygenase-2 and heat shock protein 27. These two anti-apoptotic molecules are increased and enhanced by epidermal growth factor and co-expressed with phospho-Akt in SS LSGs [27].

TLR7-related immune responses are also involved in SS pathophysiology. Zheng et al. reported that the mRNA levels of TLR7 and TLR9 in peripheral blood mononuclear cells (PBMCs) were elevated in patients with SS compared to controls, and TLR7- and TLR9-positive cells were found in the epithelial islands, lymphocytes, and ductal epithelial cells of the parotid glands in patients with SS, whereas they were limited to ductal epithelial cells in controls [28]. We analyzed the expressions of TLRs 7–9 in the LSGs of patients with SS and controls and found increased expression of TLR7 in mononuclear cells as well as ducts in patients with SS compared to controls [29]. Considering the above findings, innate immunity, especially TLRs and their downstream mediators, has been shown to play pathological roles in SS, including SG cell death.

TLR7 signaling induces type I IFNs in an IFN regulatory factor 7-dependent manner [30]. In the SGs of patients with SS, several type I IFN-regulated genes were upregulated compared to the control [31]. Upregulation of type I IFN activity was also found not to be limited to the SGs, but also systemically; Wildenberg et al. clarified increased type I IFN activity by analyzing gene expression profiling, with peripheral plasmacytoid dendritic cells being a source of this phenomenon [32]. In our analyses of TLRs 7–9 in the SGs of patients with SS, type I IFN such as IFN-α and IFN-β were also detected in mononuclear cells and ducts in the LSGs, which was more prominent in patients with SS than in controls. In addition, TLR7 stimulation induces significant upregulation of Ro52 and MHC class I in the SG epithelial cells [29]. We recently demonstrated the advancement of antigen presentation pathway of Ro52 via MHC class I molecules induced by TLR7 stimulation [33]. The upregulation of type I IFN may also have clinical significance in SS. Zheng et al. reported that serum IFN-α levels were positively correlated with ESSDAI [34].

The JAK-signal transducer and activator of transcription (STAT) pathway is an immune network with the above-mentioned TLRs, and type I IFN is an upstream signal. For example, TLR 7/8 and IFN-α were included as upstream signals of STAT1 and TLRs 7 and 9 and IFN-α were included as upstream signals of STAT3 [35]. The dysregulation of the JAK-STAT pathway is involved in various autoimmune diseases, including RA and SLE [36]. The JAK-STAT pathway is also assumed to play pathological roles in SS; Aota et al. reported that JAK1 and JAK2 are strongly expressed in ductal and acinar cells of minor SGs of patients with SS [37].

## 4. The Involvement of Long-Noncoding RNAs in SS

### 4.1. Long-Noncoding RNAs: An Emerging Class of Noncoding RNAs

In recent years, noncoding RNAs (ncRNAs), RNAs that are not transcribed into proteins, have been reported to modulate cellular functions. ncRNAs are divided into short ncRNAs and long ncRNAs (lncRNAs) according to their size. Short ncRNAs, approximately 20–30 nucleotides in length, include small nucleolar RNAs, microRNAs (miRNAs), small interfering RNAs, and PIWI-interacting RNAs [38,39]. Although newly identified ncRNAs are thought to be non-functional or considered junk DNA [40], their functions have been clarified in recent years. For instance, small nucleolar RNAs are involved in the modification of ribosomal RNAs in yeast [41,42]. In contrast, small miRNAs and small inhibitory RNAs, which have small sizes of nucleotides, affect protein-coding mRNAs [43].

lncRNAs, located in the nucleus or cytoplasm [38], are ncRNAs of 200 or more nucleotides in length [44]. They are also RNA polymerase II transcripts and are devoid of open reading frames [45,46]. The cellular mechanisms related to lncRNAs are varied: evicting proteins from chromatin, stabilizing looping and recruitment of transcriptional regulators, counteracting loop formation, recruiting proteins, scaffolding proteins, sequestering proteins or miRNAs, alternative splicing, and stabilizing mRNAs [38]. In addition to their physiological roles, aberrant expression of lncRNAs plays an important role in the invasion and metastasis of cancers, such as hepatocellular carcinoma [47]. Together with the various functions mentioned above and their high tissue specificity, lncRNAs are expected to be novel therapeutic targets, even in other types of cancers, such as non-small cell lung cancer [48] and are promising disease-specific targets for organ diseases compared to other classes of ncRNAs.

### 4.2. lncRNAs and Rheumatic Diseases

Altered expression of lncRNAs has been reported not only in organ-specific diseases, but also in rheumatic diseases such as RA, SLE, and dermatomyositis [44]. Although there are a number of studies on miRNAs that are suspected to involve in the pathophysiology of SS [49], the number of investigations of lncRNAs in SS has been limited. Investigations of increased expression of lncRNAs in SS were not reported until the late 2010s.

### 4.3. Increased Expressions of lncRNAs Found in Studies Using Blood Samples of Patients with SS

Since the late 2010s, the analyses of lncRNAs associated with SS using blood samples have been reported by many laboratories. Wang et al. collected PBMCs from patients with SS as well as healthy controls and found relative upregulation of lncRNA TMEVPG1, proportion of Th1 cells, and IFN-γ and T-box expressed in T cells in SS CD4+ cells; TMEVPG1 also had correlations with anti-Ro/SS-A antibody, erythrocyte sedimentation rate (ESR), and IgG levels. These findings suggested that TMEVPG1 might regulate the production of IFN-γ, the type II IFN which is presumed to contribute to distinct phenotypes of SS in addition to type I IFNs, by T-box expressed in T cells in the pathophysiology of SS [50]. Dolcino et al. performed analyses using PBMCs from patients with SS and healthy controls and identified three lncRNAs, LINC00657, LINC00511, and CTD-2020K17.1, as genes highly connected to the transcriptome of SS [51]. Inamo et al. found that the lncRNA LINC00487 was upregulated in B cell subsets in patients with SS, and the expression of LINC00487 was correlated with disease activity assessed by ESSDAI; LINC00487 was found to be induced by IFN-α [52], which is also regulated by TLR signaling, as discussed in Section 3 [30]. Chen Y et al. analyzed expression levels of lncRNA lnc-DC in patients with SS with immune thrombocytopenia (ITP), SS without ITP, SLE, or RA, and healthy control and found elevated expression levels of lnc-DC in patients with SS (especially those with ITP) with correlations with anti-Ro/SS-A and La/SS-B antibody, ESR, and β2 microglobulin levels [53]. Chen et al. analyzed RNA extracted from PBMCs of patients with SS and healthy controls and found that lncRNA GABPB1-AS1 and PSMA3-AS1 were upregulated in patients with SS, and GABPB1-AS1 was correlated with the percentage of B cells and IgG values [54]. Ye et al. reported that increased expression of lncRNA NEAT1 was found in peripheral T cells of patients with SS compared to healthy controls, and its expression in CD4+ T cells in patients with SS was positively correlated with disease duration, whereas it was inversely correlated with CD8+ T cells [55]. Peng et al. clarified that the lncRNA LINC00426, TPTEP1-202, CYTOR, NRIR, and BISPR are aberrantly expressed in SS, and the expression levels of the latter four are correlated with ESSDAI [56]. Amezcua-Guerra et al. analyzed the expression levels of lncRNA metastasis-associated lung adenocarcinoma transcript 1 (MALAT1) and several kinds of IFN-stimulated genes and chemokines in the PBMCs of the patients with SS as well as healthy controls after stimulation with IFN-α. They found increased expression levels of MALAT1 and several IFN-stimulated genes and chemokines, the chemokines of which were more prominent in patients with SS than in healthy controls. However, no association between serological markers of SS and expression levels of MALAT1 were found, which was thought to be due to the small sample size [57]. Joachims et al. reported the overexpression of lncRNA LINC01871 in patients with SS, suggesting that LINC01871 dysregulates T cell-mediated inflammatory pathways in SS [58]. Considering the knowledge introduced in this section, it was presumed that various lncRNAs differentially expressed in blood samples from patients with SS participated the pathophysiology of SS, the mechanism of which were in part in association with IFN-α.

### 4.4. Increased Expressions of lncRNAs in the SGs of Patients with SS

SGs are sites of anti-Ro/SS-A antibody production [59], and SG biopsy is considered as a crucial item in all classification criteria for SS. The findings of SG biopsy are not only diagnostic markers of SS but also potential predictive biomarkers of SS. The focus score, obtained by calculating the number of periductal foci of 50 or more aggregated mononuclear cells per 4 mm^2^ in the surface area of the glandular tissue [60], is reported to be associated with a higher complication rate of lymphoma [61] and interstitial lung disease [62]. Therefore, identification of increased expression of specific lncRNAs in the SGs of patients with SS may lead to disease-specific therapy for SS. However, the number of studies on the histological expression of SS-specific lncRNAs in the SGs is limited compared to that in blood samples. Shi et al. analyzed expression profiles of lncRNAs in the LSGs of patients with SS and healthy controls by real-time polymerase chain reaction, and revealed that eight lncRNAs (ENST00000420219.1, ENST00000455309.1, n336161, NR_002712, ENST00000546086.1, lnc-UTS2D-1:1, n340599, and TCONS_l2_00014794) were significantly upregulated in SS, each of which was correlated with disease duration or biomarkers such as ESR, rheumatoid factor, and serum β2 microglobulin levels [63]. This study showed the importance of upregulated lncRNAs in the SGs contributing to SS pathophysiology; however, the expression of lncRNAs in the LSGs has not been studied histologically. Therefore, we conducted the first study to analyze the intracellular localization of SS-related lncRNAs in the LSGs.

Noncoding repressor of the nuclear factor of activated T cells (NFATs) (NRON) is a lncRNA that regulates the activity of NFATs, which has been suggested to be involved in the pathophysiology of SS [64] by altering its intracellular localization [65]. We hypothesized that NRON is a candidate disease-related lncRNA for SS and that substances which consist of the NFAT-regulation mechanism including NRON, are aberrantly expressed in the SS LSGs. Therefore, we conducted a study to clarify the expression of NRON and related substances in the LSGs of SS and their relationship to the clinical parameters of SS. We found NRON expression in the nuclei of the LSGs, strongly in ducts and weakly in alveoli. Particularly prominent expression was detected in infiltrating cell areas of the SS LSGs. We then analyzed the expression of NFATc1, as well as its dominance in whole infiltrating cell areas, and found that the NFATc1-positive area/infiltrating cell area was positively correlated with the size of the infiltrating cell area and focus score, suggesting that both the size of the infiltrating lymphocyte area and the number of infiltrating lymphocyte areas are important for NRON-NFATc1 activation. These findings suggested that NRON participates in the pathophysiology of SS along with NFATc1 [66]. LncRNAs introduced in this article as aberrantly expressed in SS are summarized in Table 1.

## 5. Future Therapeutic Perspectives on SS Based on Viral Infection, TLRs, lncRNAs, and Related Signals

Considering potential roles of viruses on SS, interventions against viral infections may be beneficial in the prevention and/or treatment of SS. Efforts to develop HTLV-1 vaccines have been made [67]; however, whether HTLV-1 vaccination would be beneficial in decreasing SS needs to be analyzed in the future. Nucleoside/nucleotide analog therapy against HBV has been reported to lower the risk of SS in patients with HBV infections [68]. However, IFN-based anti-HCV therapy has not been shown to contribute to a lower risk of SS [69].

Based on the pathogenic mechanism of SS described in Section 3, TLRs and their downstream molecules may be novel potential therapeutic targets in SS. Hydroxychloroquine (HCQ) inhibits TLR signaling by accumulating in and altering the pH of endosomes and directly binding to nucleic acids, which blocks TLR signaling [70]. HCQ ameliorates microbiota dysbiosis in patients with SS [71]. For pregnant women with autoimmune diseases, including SS, treatment with HCQ has been reported to improve perinatal outcomes, and early introduction by 3–6 months before pregnancy is recommended due to its slow-acting characteristics [72]. However, administration of HCQ to patients with SS has not been proven to improve sicca symptoms, pain, and fatigue in a previous randomized clinical trial, which concluded that further long-term analyses are needed [73]. As for the eyes, there was concern that treatment with HCQ would exacerbate microvascular alterations in patients with SS, which suggested the necessity of alerting patients with SS against the potential adverse effects of HCQ [74]. Calcineurin inhibitors such as cyclosporine and tacrolimus were reported to induce inhibition of TLR signaling in the PBMCs of the patients who underwent liver transplantation [75]. Tacrolimus, a 23-membered-ring macrolide [76], was reported to improve refractory immune thrombocytopenia associated with SS [77].

As discussed in Section 3, type I IFNs are upregulated in association with TLR signaling. Type I IFNs are well-studied therapeutic targets in other rheumatic diseases, particularly SLE. Patients with SLE are often positive for anti-Ro/SS-A antibody, the production of which was presumed to be affiliated by IFN-α [22]. Anifrolumab, a fully human anti-IFN-α monoclonal antibody, has proven beneficial for the treatment of refractory SLE [78]. Anti-IFN-α monoclonal antibody might be beneficial for improving a part of the pathophysiology of SS, however, no studies have investigated the therapeutic effects of anifrolumab on SS.

Vitamin D is a secosteroid ingested by the human body or synthesized in the skin in response to ultraviolet B [79]. Recently, the therapeutic effects of vitamin D, which has modulatory effects on innate immunity, have attracted attention. Deficiency and therapeutic effects of vitamin D have been observed in various rheumatic diseases such as RA and SLE, and the ameliorating effects of vitamin D on rheumatic diseases are, in part, presumably attributed to its suppressive effects on the expression of TLRs [80]. According to the study cited in Section 3 by Zheng et al., significantly lower concentrations of serum 25-OH vitamin D3 were found in patients with SS than in healthy controls. Furthermore, a negative correlation between serum 25-OH vitamin D3 concentrations and ESSDAI, a positive correlation between serum IFN-α levels and ESSDAI, and a negative correlation between serum 25-OH vitamin D3 concentrations and serum IFN-α levels were found [32]. These findings indicate the suppressive effects of vitamin D3 on IFN-α and the possibility that vitamin D supplementation might help relieving symptoms of SS. Rashidmayvan et al. investigated the association between vitamin D and lncRNA expression in patients with metabolic syndrome and found an inverse association between vitamin D and MALAT1 levels [81]. Considering the potential role of MALAT1 in SS [57], vitamin D may improve a part of the pathophysiology of SS through MALAT1 inhibition. The clinical application of lncRNA-targeted therapy for SS-related sicca symptoms is yet to be studied. However, the associations between hyposalivation and lncRNA expression profiles have been studied in the submandibular glands of hypertensive rats, suggesting that lncRNA-targeted therapy may be a novel therapeutic option for hyposalivation [82]. Further studies are needed to apply lncRNAs that are upregulated in SS, such as MALAT1 and NRON, to SS-specific lncRNA-based gene therapy specific to SS.

As described in Section 1, JAKis are effective against refractory rheumatic diseases, especially RA. The therapeutic effects of JAKis have also been reported in other rheumatic diseases, such as SLE, in which dysregulation of the JAK-STAT pathway is involved [36]. Currently, several classes of JAKis, such as tofacitinib, baricitinib (BAR), peficitinib, filgotinib (FLG), and upadacitinib, are approved for the treatment of autoimmune diseases, including RA, and each JAKi has its own characteristic point of action different from other classes. Tofacitinib, a JAKi with a high selectivity on JAK1 and JAK3, was reported to suppress production of type I IFNs including IFN-α [83,84], and BAR, another JAKi with a high selectivity on JAK1 and JAK2, was also found to inhibit production of type I IFNs [85].

**Figure 1:**
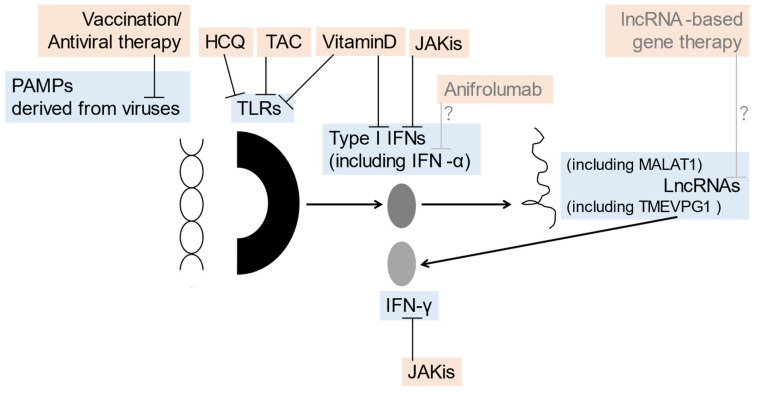
The association between viral infections, TLRs, lncRNAs, and related potential therapeutic options of SS discussed in this article: prevention/therapeutic targets (blue) inhibited by prevention/therapeutic options (orange) such as vaccination/antiviral therapy, HCQ, vitamin D, JAKis, anifrolumab, and lncRNA-based gene therapy are shown. HCQ, hydroxychloroquine; IFN, interferon; JAKis, Janus kinase inhibitor; lncRNA, long-noncoding RNA; MALAT1, metastasis-associated lung adenocarcinoma transcript 1; PAMPs, pathogen-associated molecular patterns; TLRs, Toll-like receptors.

Recent experimental findings on JAKis in the SGs have suggested that JAK inhibition may also be applicable to the treatment of SS. Aota et al. revealed that BAR suppressed IFN-γ-induced expression of CXCL10 in human SG ductal-cell clone in a dose-dependent manner and inhibited the IFN-γ-induced STAT1 and STAT3 phosphorylation [35]. Lee et al. reported that FLG, a selective inhibitor of JAK1, suppressed IFN-related genes and B cell-activating factors in the human SG epithelial cells of patients with SS [86]. The clinical effects of BAR on SS, which were reflected as an improvement in ESSDAI, were observed in a relatively small patient group with SS [87], which has yet to be clarified in a randomized controlled study [88]. In contrast, the therapeutic effects of FLG on active SS, another class of JAKi, have already been studied in a randomized double-blind, placebo-controlled study, which did not reach statistical significance [89]. The associations between viral infections, TLRs, lncRNAs, and related signals, as well as the potential therapeutic options for SS discussed in this article, are summarized in Figure 1.

## 6. Conclusions

According to previous reports introduced in this review, viral infections, TLRs, and lncRNAs may play pivotal roles in the pathophysiology of SS. Viral infections inducing TLR activation and immune signals related to TLRs, such as type I IFNs and the JAK/STAT pathway, have been studied as potential therapeutic targets for SS. In addition, lncRNAs may be potential biomarkers in which molecular targeted therapy could be applied for the treatment of SS. Nevertheless, current knowledge about SS on lncRNAs is still limited compared to that on miRNAs, and the amount of data in vivo is limited compared to that in vitro, that is, problems such as unexpected adverse effects and dose-dependent analysis have to be overcome before clinical applications. Further studies on viral infections, TLRs, and lncRNAs associated with SS are expected to contribute to the development of specific diagnostic procedures and therapies for SS.

## Figures and Tables

**Table 1:** LncRNAs that have been found to be associated with SS.

LncRNA	Samples Used to Analyze Expression Levels	Reference
TMEVPG1	PBMCs	[50]
LINC00657	PBMCs	[51]
LINC00511	PBMCs	[51]
CTD-2020K17.1	PBMCs	[51]
LINC00487	PBMCs	[52]
lnc-DC	PBMCs	[53]
GABPB1-AS1	PBMCs	[54]
PSMA3-AS1	PBMCs	[54]
NEAT1	PBMCs	[55]
LINC00426	PBMCs	[56]
TPTEP1-202	PBMCs	[56]
CYTOR	PBMCs	[56]
NRIR	PBMCs	[56]
BISPR	PBMCs	[56]
MALAT1	PBMCs	[57]
LINC01871	PBMCs	[58]
ENST00000420219.1	LSGs	[63]
ENST00000455309.1	LSGs	[63]
n336161	LSGs	[63]
NR_002712	LSGs	[63]
ENST00000546086.1	LSGs	[63]
lnc-UTS2D-1:1	LSGs	[63]
n340599	LSGs	[63]
TCONS_l2_00014794	LSGs	[63]
NRON	LSGs	[66]

LSGs, labial salivary glands; PBMCs, peripheral blood mononuclear cells.

## Data Availability

Not applicable.
